# Spring constant and sensitivity calibration of FluidFM micropipette cantilevers for force spectroscopy measurements

**DOI:** 10.1038/s41598-019-46691-x

**Published:** 2019-07-16

**Authors:** Ágoston G. Nagy, Judit Kámán, Róbert Horváth, Attila Bonyár

**Affiliations:** 1 Nanobiosensorics Laboratory, Institute of Technical Physics and Materials Science, Centre for Energy Research, Hungarian Academy of Sciences, Budapest, Hungary; 2 Department of Electronics Technology, Budapest University of Technology and Economics, Budapest, Hungary

**Keywords:** Cell adhesion, Characterization and analytical techniques, Scanning probe microscopy, Atomic force microscopy

## Abstract

The fluidic force microscope (FluidFM) can be considered as the nanofluidic extension of the atomic force microscope (AFM). This novel instrument facilitates the experimental procedure and data acquisition of force spectroscopy (FS) and is also used for the determination of single-cell adhesion forces (SCFS) and elasticity. FluidFM uses special probes with an integrated nanochannel inside the cantilevers supported by parallel rows of pillars. However, little is known about how the properties of these hollow cantilevers affect the most important parameters which directly scale the obtained spectroscopic data: the inverse optical lever sensitivity (InvOLS) and the spring constant (*k*). The precise determination of these parameters during calibration is essential in order to gain reliable, comparable and consistent results with SCFS. Demonstrated by our literature survey, the standard error of previously published SCFS results obtained with FluidFM ranges from 11.8% to 50%. The question arises whether this can be accounted for biological diversity or may be the consequence of improper calibration. Thus the aim of our work was to investigate the calibration accuracy of these parameters and their dependence on: (1) the aperture size (2, 4 and 8 µm) of the hollow micropipette type cantilever; (2) the position of the laser spot on the back of the cantilever; (3) the substrate used for calibration (silicon or polystyrene). It was found that both the obtained InvOLS and spring constant values depend significantly on the position of the laser spot. Apart from the theoretically expectable monotonous increase in InvOLS (from the tip to the base of the cantilever, as functions of the laser spot’s position), we discerned a well-defined and reproducible fluctuation, which can be as high as ±30%, regardless of the used aperture size or substrate. The calibration of spring constant also showed an error in the range of −13/+20%, measured at the first 40 µm of the cantilever. Based on our results a calibration strategy is proposed and the optimal laser position which yields the most reliable spring constant values was determined and found to be on the first pair of pillars. Our proposed method helps in reducing the error introduced via improper calibration and thus increases the reliability of subsequent cell adhesion force or elasticity measurements with FluidFM.

## Introduction

The fluidic force microscope (FluidFM) system was created based on the principles of the atomic force microscope (AFM)^1^: a nanofluidic channel is attached to a refillable fluid reservoir, which is introduced into an AFM cantilever regulated by a pressure control system^2^.The special microfabrication technology^3^ of these hollow cantilevers allows the user to dispense or collect fluids in the femtoliter scale enabling wide functionallity^4^. The FluidFM setup has been used for 2D and 3D printing^4,5^, colloidal probe technique^6,7^, injection or extraction of liquids into/from living cells^2,8^ and single-cell force spectroscopy (SCFS)^9–17^. FluidFM cantilevers are available in two different constructions. They are equipped either with a hollow pyramid or a flat aperture with various sizes designed specifically for the given application areas^16^. By using the FluidFM system several previously existing AFM techniques became much easier to perform. As an example, although SCFS measurements can be carried out with a cell attached to a functionalized AFM cantilever^18,19^, such methods are time consuming, elaborate and require both expertise and patience. The FluidFM micropipette cantilevers facilitate such procedures with much easier attachment of the spread cells to the cantilever’s outlet^15^. During SCFS a characteristic force-distance (F-D) curve can be obtained from which properties such as the maximal adhesion force, adhesion energy and step-like events displaying the rupture of individual receptor-ligand interactions anchoring the cells to the substrate can be calculated^20^. This type of spectroscopic data carries information regarding the cellular state, and the cells’ attachment to engineered or natural surfaces may reflect on their behavior represented by the F-D curves. Furthermore, the elastic property of the cells is another parameter that influences cellular behavior, migration, differentiation, and polarization, characterized by the Young’s modulus. As an example, cellular elasticity has an effect on the regulation of cancer cells and their transformation to malignant cells^21^. Cancer cells also have influence on the regulation of the environment, because they cause the disintegration of the endothelial barrier by softening the endothelial cells^22^. Cellular stiffness can be probed with various methods yielding diverse results^23^, however, AFM is the golden standard when it comes to cell elasticity determination. The Young’s modulus can be obtained by probing the cell with a sharp, spherical, spheroconical, conical, or flat cantilever, and fitting the measured point-spectroscopy curves with a contact-mechanical model^24^. The FluidFM micropipette cantilever has a similar shape to flat AFM cantilevers, thus by using the appropriate model for fitting (e.g. Herz-Sneddon model^25^) it can be utilized for the same purpose.

It is well-known in classical AFM methodology, that the precise calibration of the spring constant^25,26^ and optical lever sensitivity^24^ of the used cantilever is essential to gain reliable data with the above mentioned methods. Since the FluidFM micropipette cantilevers have a unique, non-standard structure compared to usual AFM cantilevers^2,15,25^ questions about possible differences or even difficulties regarding their calibration rightfully arise. Until now only few researches have published SCFS data^9–15,27^ using the FluidFM system with its micropipette cantilever and presented results regarding a variety of cell types and adhesion forces. Some of these results are collected in Table 1, showing that the FluidFM technology has a great potential in obtaining large quantities of data and yielding statistically relevant biological information. The standard error of the collected cellular adhesion experiments range from 11.8 to 50%, which may be accounted for the natural variation in biological systems or biological diversity. However, as we will demonstrate later, improper determination of either the spring constant or the sensitivity of the cantilever by neglecting important aspects of their calibration could also introduce such, or even higher errors into the measured data. As it can be seen in Table 1, in some experiments users only applied the nominal value of the spring constant without any calibration.

**Table 1:** Examples for the application areas of force-distance curves measured by FluidFM, collected from the literature.

Application type	Experiment	Used InvOLS and *k* calibration	Rupture force	Reference
Grabbing the spread cells or bacteria with the FluidFM micropipette cantilevers and detaching them from the surface to measure adhesion forces	Measuring the effect of electric current on cellular adhesion using mouse myoblast (C2C12) cells.	InvOLS was recalibrated before all experiments on the substrate. Spring constant was calibrated with the Sader method. Exact values are not given.	The maximal adhesion force in control conditions on indium tin oxid coated glass slides was 520 nN ± 67,6 nN	^9^
	Measuring cellular adhesion using mouse myoblast (C2C12) cells on different type of substrates.	Spring constant ranged between 1.7 and 2.3 Nm^−1^. InvOLS was calibrated before each measurement on a cell free spot.	Median values on RGD presenting serum, covalent and non-covalent surfaces respectively: 236 nN, 409 nN, 425 nN; No detailed description of the measured errors.	^10^
	Detachment of individual cells and cells from monolayer from glass (L929 Fibroblasts) and gelatin coated glass (Human umbilical artery endothelial cells).	Spring constant was calibrated with the thermal noise method; InvOLS with Cytosurge’s built-in software. Exact values are not given.	*Individual cells (mean values):* L929: 234 nN HUAECs: 805 nN *Cells from monolayer (mean values):* L929: 232 nN HUAECs: 1170 nN	^11^
	Measuring HeLa and HEK cells adhesion on glass and fibronectin in culture and room temperature environments.	Spring constant was calibrated with the Sader method and ranged between 1.9 and 2.7 Nm^−1^. InvOLS calibration and values not presented.	*Mean values on glass:* HeLa cells: 473 ± 127 nN HEK cells: 33 ± 9 nN *Mean values on fibronectin:* HeLa cells: 593 ± 70 nN HEK cells: 53 ± 15 nN	^12^
	Detachment of Escherichia coli and Streptococcus pyrogenes bacteria strains from polydopamin treated surface.	Cantilevers with nominal spring constants of 2.5 and 0.2 Nm^−1^ were used. InvOLS calibration and values not presented.	Force values are following a Gaussian distribution with mean values around 6–8 nN in the range of 0–14 nN.	^13^
	Detachment of neural cells from glass slides functionalized with fibronectin.	The details of spring constant and InvOLS calibration and not discussed.	Force to detach neural cells: 1000 ± 300 nN	^15^
	Detachment of Human umbilical vein endothelial cells (HUVECs) from gelatin coated gratings with 100, 400 and 1000 nm depth and 1000 nm width.	The spring constant was determined to be around 1.8 Nm^−1^ with the Sader method. InvOLS calibration and exact values are not presented.	Mean adhesion forces on substrates with different topology: Flat control surface −619 ± 70 nN 100 nm deep grating −1113 ± 86 nN 400 nm deep grating −860 ± 59 nN 1000 nm deep grating −598 ± 123 nN Treatment with myosin-II inhibitor Blebbistatin on the control surface resulted in the decrease of adhesion force: 295 ± 44 nN	^17^
Application type	**Experiment**		***Used InvOLS and*** **k** **calibration**	**Reference**
Colloidal spectroscopy	Concanavalin-A coated colloidal particles were adsorbed on human embryonic kidney cells. The particles were detached from the cells, which enabled the measurement of the interacting forces between them. The adhesion force was ~60 nN between the particles and the cells, and individual cells showed ~20 nN adhesion force on the glass petri dish.		The spring constant was determined with the Sader method and resulted between 0.5 to 3 Nm^−1^. InvOLS was calibrated each time the medium of the experiment was changed.	^6^
	Reversible immobilization of functionalized silica beads onto the FluidFM cantilever, adhering bacteria and measuring hydrophobic interaction of the bacteria from leaves. 28 bacterial strains have been used for colloid particle-bacteria surface adhesion measurement. More than 700 FD-curves were recorded, the highest force values are around 50 nN of the members from Gammaproteobacteria.		The nominal spring constant value for micropipette cantilevers was used as 0.2 Nm^−1^. InvOLS was recalibrated after each bead exchange, but exact values are not given.	^33^
	Force evaluation of different particle sizes grabbed by FluidFM micropipette and nanopipette cantilevers. Silica particles with diameters of 0.5 µm, 1 µm and 4.3 µm were used.		The nominal spring constant values were used for micropipette (0.3 and 2 Nm^−1^) and nanopipette (0.6 Nm^−1^) cantilevers. Exact *k* and InvOLS values are not given.	^7^
	Polyanionic and polycathionic recombinant spider silk protein was used to prepare colloidal particles, for testing the biofunctionality of the material with FluidFM.		The nominal spring constant values were used for micropipette (0.2 Nm^−1^) cantilevers. “InvOLS was determined in a symmetric system between two silica particles.” Exact k and InvOLS values are not given.	^34^

(A) Cellular adhesion experiments on different cell types and experimental conditions. (B) Colloidal spectroscopy and force analysis.

In our paper we aim to answer the following questions. (1) Does the special structure of the FluidFM cantilevers (hollow rectangular slab with embedded channels, pillars and a circular aperture at one end) has any effect on the calibration of the spring constant or lever sensitivity? Since both of these parameters are measured with the laser beam reflection based optical detector, any differences compared to classical AFM cantilevers are expected to be discernable in this context, which leads to the next few questions. (2) Does the spring constant calibration depend on the position of the directed laser spot; and (3) does this laser position on a structurally inhomogeneous cantilever affect the measured lever sensitivity in any way, as opposed to what would be expected for a homogenous slab? (4) Does the aperture size (2, 4 and 8 µm) at the end of the micropipette cantilever affect the calibration in any way? Our last main question is related more to the experimental conditions. The FluidFM BOT system is designed in a way that its sample holder could incorporate standard sized plates (such as a Corning 6-welled polystyrene plate), which can contain the investigated cell cultures. For the calibration of lever sensitivities in practice an ideally hard and flat sample should be used (such as piece of silicon wafer for example) to avoid the deformation of the indented surface, which would lead to false obtained sensitivities. Using the polystyrene sample holder plate for sensitivity measurements would be more convenient, however, since the elastic modulus of polystyrene is nearly two orders of magnitude smaller than that of silicon, it is rightful to ask that, (5) would using a polystyrene plate instead of silicon introduce any significant error during the calibration of lever sensitivity?

These five main questions will be investigated experimentally and discussed in detail in the following sections.

## Principles of Operation and Theoretical Considerations

**Figure 1:**
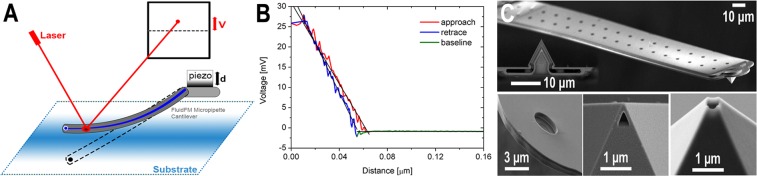
(**A**) Illustration of the laser beam reflection based optical setup of the FluidFM system. (**B**) A sample point-spectroscopy curve, performed as calibration with the FluidFM of the polystyrene substrate (corresponding to the 2 μm aperture, measured at the reference (0) position, as defined as the laser beam positioned directly above the first pair of pillars). (**C**) Scanning electron microscope images of various FluidFM cantilevers showing the parallel rows of pillars holding the microfluidic channel inside the hollow cantilever. The smaller images represent the types of apertures used for different applications from left to right: micropipette, rapid prototyping, nanopipette head. The schematics of the micropipette cantilevers are presented later in Fig. 3. (**C**) is curtesy of Tomaso Zambelli^35^.

The FluidFM system uses the classical laser beam reflection based method to measure the deflection of the cantilever^1,2^: a focused laser beam is directed onto the reflective back side of the nanofluidic cantilever, the reflected beam hits a two segmented position-sensitive photodetector (PSPD), which senses the movement of the laser beam caused by the bending of the cantilever and produces a signal corresponding to the interactions between the cantilever’s tip and the surface (Fig. 1A). Note that since the FluidFM uses a two segmented detector it is not possible to measure torsional information or lateral forces with this system. In this manner the interactions during an SCFS measurement are primarily characterized by the measured voltage function (*U*(*t*) [V]).The force between the cell and the cantilever can be calculated by using Hooke’s law (Eq. 1), where *k* [N/m] is the spring constant of the cantilever, *δ*(*t*)[m] is its deflection, calculated by scaling the measured voltage(*U*(*t*),[V]) with the inverse optical lever sensitivity (InvOLS, [m/V]) of the cantilever^28^.

1 $${F}_{c}(t)=k\ast \delta (t)=k\ast U(t)\ast InvOLS$$

The InvOLS of a cantilever is usually determined by performing point-spectroscopy on an ideally hard and flat surface, like a silicon wafer. In this case the deformation of the surface can be neglected, thus pressing the probe to the surface by lowering the piezo stage in the normal direction will result only in the deflection of the cantilever, which causes a linear signal in the PSPD as functions of the piezo position producing the so called sensitivity approach curve (Fig. 1B), where the PSPD output [V] is plotted against the position [µm]. InvOLS is usually calculated as the slope of this linear approach curve as in Eq. 2, where *∆z* is the piezo movement.

2 $$InvOLS=\frac{{\rm{\Delta }}z}{{\rm{\Delta }}U}$$

Out of the several methods^26,29^ which could be used for the determination of the cantilever’s spring constant, the software of the FluidFM uses the Sader method, discussed in detail elsewhere^25,29,30^. Besides predefined (and embedded) material and geometrical properties, the Sader method relies on the measured position and quality factor (*Q*) of the first, fundamental resonance frequency (*ω*_R_) of the cantilever^26^. The original Sader model is presented in Eq. 3, where *w* and *L* are the width and length of the cantilever, while *ρ* and Γ_i_ are the density and imaginary part of the dimensionless hydrodynamic function of the fluid (such as air) evaluated at the resonant frequency^29^.

3 $$k=0.1906\,\rho {w}^{2}\,LQ{{\rm{\Gamma }}}_{i}({\omega }_{R}){\omega }_{R}^{2}=0.1906\,\rho {w}^{2}L{{\rm{\Gamma }}}_{i}({\omega }_{R}){\omega }_{R}^{3}FWH{M}^{-1}$$

*Q* and *ω*_R_ are obtained by measuring the thermal noise spectrum of the unloaded cantilever in air and finding the position and full width half maximum (FWHM) of the resonance peak by fitting a Lorentzian function on the raw spectrum. A weakness of the Sader method lies in the precise determination of the quality factor, and since the optical detector is used to measure the thermal noise spectrum, parameters which affect the optical reflection based setup are expected to influence the determination of the spring constant as well.

One of the main applications of SCFS is to measure cellular adhesion based on the retrace part of a force-distance curve (*F*_c_(*d*)). As can be seen based on Eq. 1, the spring constant and the InvOLS are key parameters, which directly scale the voltage signal (*U*(*t*)) measured by the PSPD, so any error introduced by the inadequate calibration of these parameters will be directly reflected in the measured adhesion forces. In the other major application of SCFS the elastic properties of the cells are measured by fitting a contact-mechanic model on the approach part of the force-distance curve, where the deformation of the cell is apparent in a characteristic way. The Hertz model is the most commonly used to evaluate the curves and determine of the Young’s modulus (*E*) of the cells^31,32^. A rearranged form of the model is written in Eq. 4, where *z* is the actual piezo position, *z*_0_ is the position of the piezo at the contact point, *k* is the spring constant, ν is the Poisson ratio, *R* is the tip radius, and *δ*(*t*) is the relative deflection of the cantilever.

4 $$k\delta (t)=\frac{4E\sqrt{R}}{3(1-{\nu }^{2})}{(z(t)-{z}_{0}-\delta (t))}^{3/2}$$

**Figure 2:**
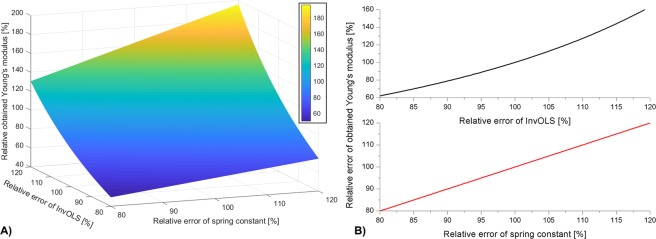
(**A**) The relative error of Young’s modulus calculated from a force-curve with the Hertz model (Eq. 4) is plotted against the relative error of spring constant and InvOLS determination. (**B**) Cross-sections of the 3D plot at 100% relative spring constant (top) and 100% relative InvOLS (bottom) respectively.

Since *δ*(*t*) is derived by scaling the measured *U*(*t*) with InvOLS (Eq. 1), we can see that the Young’s modulus implicitly depends on InvOLS, while also depending linearly on the spring constant. Based on Eq. 4, in Fig. 2 we visualized the relative error of the determined Young’s modulus introduced by the error of InvOLS and spring constant determination. Since *E* cannot be explicitly expressed as a function of InvOLS due to the scaling of *U*(*t*) and subsequent model fitting on the scaled data, a deflection-curve was artificially generated for 60 nm piezo movement with the following nominal parameters: *k* = 2 N/m, *E* = 50 MPa, InvOLS = 2.3 μm/V, ν = 0.5, *R* = 20 nm. Subsequently Eq. 4 was fitted on the generated deflection-curve, by varying *k* and InvOLS as input parameters. As can be seen, in the ±20% error range of *k* and InvOLS the relative error of the calculated Young’s modulus is in the −50%/+100% range, which can be considered unacceptably high for several applications.

Conclusively there are three main parameters directly measured by the FluidFM (InvOLS, *ω*_R_ and FWHM), which affect the calculated spring constant, force and Young’s modulus (*k*, *F*_c_ and *E*). Equations 1–4 show the explicit dependence of the calculated quantities from these measured parameters, but in some of the cases, there is also an implicit dependence (e.g. because scaling model fitting is involved). These relations will be experimentally investigated in the next sections. For more information also see the Supplementary Information section.

## Experimental

**Figure 3:**
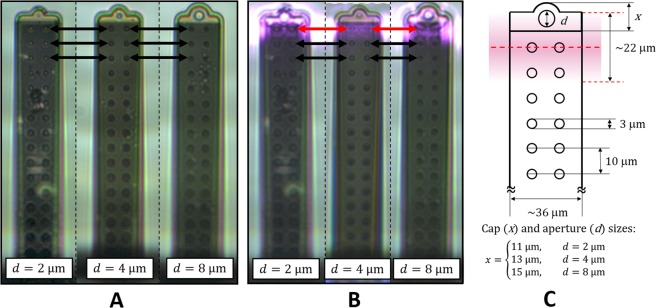
(**A**) Optical microscopy images (exported from ARYA) from the three cantilevers with different aperture sizes. (**B**) The same cantilevers with the laser spot positioned directly over the first pair of pillars (indicated by red arrows), which is used as the reference (0) position in all of the experiments. (**C**) Illustration of this assigned reference position with the geometrical parameters of the cantilevers (*d*: aperture size, *x* cap size). Different aperture sizes require different cap sizes, as indicated in the label. The center of aperture opening is always located 192 μm from the base of the cantilever.

In all experiments the FluidFM BOT setup from Cytosurge AG. (Glattbrugg, Switzerland) was used, which includes an inverted optical microscope (Zeiss Axio Observer.Z1, Carl Zeiss AG, Oberkochen, Germany), an XY sample stage and an automated Z stage serving as the measurement head combined with a pressure control system. With a mounted FluidFM micropipette the Z stage could be moved independently from the XY stage with a resolution of 1 nm. To position the probe over the polystyrene sample holder the inverted optical microscope was used. For the Si wafer surface we relied on the force-feedback mechanism of the system. Silicon nitride micropipettes with 2 μm, 4 μm and 8 μm aperture sizes (Fig. 3) and with 2 N/m nominal spring constant were purchased from Cytosurge AG and used for the measurements.

InvOLS measurements were carried out in Milli-Q water on two substrates: (1) a 6-well non-coated polystyrene plate for tissue culture applications (Corning, New York, USA); and (2) a Piranha solution cleaned silicon wafer, which was superglued to the bottom of one of the wells. The Corning 6-well polystyrene plate was chosen because many applications with the FluidFM involve mammalian cell lines cultured in these plates. FluidFM micropipettes were filled with Milli-Q water under sterile conditions to avoid contamination getting into the nanofluidic channel of the cantilever and were mounted to the Z stage. The whole system and experimental processes were controlled by the ARYA software, which is specially designed to perform experiments with the FluidFM BOT using a 2 segmented PSPD (Fig. 1A). With the ARYA software it is possible to define the position of the laser spot on the back of the cantilever. Two sets of experiments were performed: first, the position of the laser spot was changed in 10 μm steps from tip to base, while in the second set of experiments 1 μm steps were used. As can be seen in Fig. 3, the geometrical parameters of the used cantilevers vary: the cap size at the tip of the cantilever (around the aperture) is a function of the aperture size, since center of the aperture is located exactly at 193 μm distance from the base of the cantilever larger apertures require larger caps compared to smaller aperture sizes (Fig. 3C). This variation does not change important and fixed parameters of the cantilever (e.g. length, pillar position, width, etc.). To have a common reference point we decided to assign the zero position as illustrated in Fig. 3B,C. The laser illuminates an approximately 22 μm section of the cantilever and expected to have a Gaussian intensity distribution. In the zero position the midline (intensity maximum) of the laser spot matches the midline of the first pillars. These exact positions were determined based on optical microscopy images, by using image processing. The estimated precision of the position determination is around 1 μm, for all data presented in the paper.

The InvOLS values were determined by ARYA automatically, which are calculated based on the linear fit on the approach phase of the deflection curve, starting at the contact point between the cantilever and the surface (Fig. 1B). Around 2400 deflection-curves have been recorded in total and every fit on each curve was reviewed manually. Around 5% of all measurements were considered as non-reliable InvOLS values and were discarded. The standard deviations given in the figures for the InvOLS experiments were calculated based on 10 deflection-curves in every position. In every measurement position the spring constant was also measured. The spring constant values are also automatically determined by ARYA using the Sader method. Unlike to InvOLS, parameters were kept constant, and the repeated determination of the spring constant at a given laser position has an error below 3%.

## Results and Discussion

### Fluctuations in *k* and InvOLS as functions of the laserspot position

**Figure 4:**
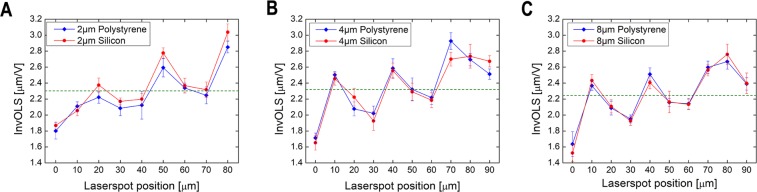
The obtained InvOLS values measured in 10 µm steps on polystyrene and silicon substrates as functions of the laser position for FluidFM cantilevers with (**A**) 2 µm, (**B**) 4 µm, and (**C**) 8 µm apertures. The data points represent the average and standard deviation of 10 consecutive measurements in every point. The dashed lines indicate the average value for a given cantilever considering all data.

First, we investigated the effect of laser beam spot position on the measured InvOLS values for all cantilevers. Figure 4 presents the results obtained on both polystyrene and silicon substrates. It has to be noted, that theoretically the obtained InvOLS values should show a monotonously increasing tendency^24^ as functions of the spot’s position, from the tip to base, consistently with the cantilevers deformation along its length. Instead, we observed a periodic fluctuation, which is superposed on the increasing trend along the cantilevers’ length. This fluctuation can clearly be associated with the irregular inner structure of FluidFM micropipette cantilevers, which differ from regular, slab-like AFM cantilevers^25^ with characteristic monotonic lever sensitivity^24,28^ curves. The FluidFM cantilevers incorporate a nanofluidic channel supported by parallel rows of pillars to increase the stability^25^. It can be established, that the stresses evolving in the hollow cantilever and its subsequent deformation caused by indentation will not be homogenous along its length. The pillars have a diameter of 3 µm and are evenly spaced in every 10 µm (see Fig. 3). However, the laser spot’s diameter is large enough (around 22 µm with a Gaussian intensity distribution) to illuminate more than one pair of pillars at the same time. The local deformation of the cantilever, the intensity distribution of the laser spot and possible interference effects together determine the measured deflection signal from one spot, which cannot be easily predicted or calculated. However, two important facts can be concluded based on the results in Fig. 4: (1) the fluctuation of InvOLS as functions of laser position is consistent and repeatable; the measurements independently performed on the two different substrates were consistent with each other. (2) The calibration on the polystyrene plate did not result in a significant difference compared to the ideally flat and hard Si wafer. This confirms that the plastic plate can be used for calibration purposes as well.

An interesting aspect of the curves in Fig. 4 is that the periodicity of fluctuation does not follow directly the 10 µm periodicity of the pillars in the channel. To investigate this in more detail we repeated the experiments and measured the InvOLS with a finer 1 µm resolution, using only the PS plate and focusing only on the most important area of the cantilever: closest to the tip, where the majority of users position their laser. Again, the reference position (0 µm) is selected to be directly over the first pair of pillars (see Fig. 3B,C). The negative positions mean that the intensity maximum of the spot was positioned between the tip of the cantilever and the first pair of pillars (Fig. 3). Positioning the laser directly on the apertures will result in increased interference and noise, especially for cantilevers with larger aperture sizes, yielding consistently unreliable sensitivity values (these values were discarded).

**Figure 5:**
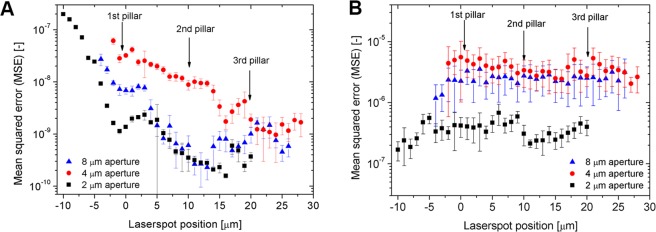
(**A**) The obtained InvOLS values measured in 1 µm steps on the polystyrene substrate with the three FluidFM cantilevers. The curves represent a 5^th^ grade polynomial fit on the datasets. (**B**) The spring constant measured in 1 µm steps in air with the three FluidFM cantilevers, as functions of the laser spot position.

It can be seen in the results of Fig. 5A that although the three cantilevers with different aperture sizes resulted in different absolute sensitivities, their trends – highlighted by the polynomial fit on the datasets – are running together. The InvOLS has a definite local minimum at the first pair of pillars (0 µm), in other words, the deformation of the cantilever is the highest here. The next local minimum is around the third pillars, between these two, the inverse sensitivity has a local maximum around the second pair of pillars.

**Figure 6:**
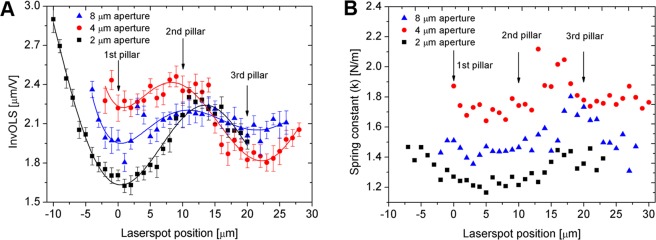
(**A**) The noise levels calculated as mean squared error (MSE) of linear fit on the baseline of a force-curve (see Fig. 1B). (**B**) The same MSEs, measured on the linear indentation (approach) section of the force-curve, which was used for InvOLS calculations.

It is important to emphasize, that the observed sinusoidal oscillation trend in the InvOLS curves (Fig. 5A) is connected to the structural characteristics and deformation of the FluidFM probes and is independent from any noise superposed on the deflection-curves. It can be seen based on the sample deflection-curve presented in Fig. 1B that a noise originating from laser interference is present. As mentioned before, this effect is the strongest above the apertures and seemingly decreases along the length of the cantilevers. To quantify this noise we calculated the mean squared error (MSE) of linear fitting both for the baseline (Fig. 6A) and also for the linear indentation section (Fig. 6B) of the deflection-curves (see Fig. 1B for the illustration). The results indicate that the noise level drops several orders of magnitude along the cantilever (from the aperture to the third pillar), but only on the baseline. The noise superposed on the linear part of the deflection-curves is independent from the laser spot’s position along the cantilever, which confirms our conclusions regarding Fig. 5A.

With the same resolution the spring constant was measured, as evaluated by the built-in Sader method of ARYA. The results are given in Fig. 5B. Again, the curves show similar tendencies (e.g. a definite peak around 15 µm), but their meaning needs further investigation.

**Figure 7:**
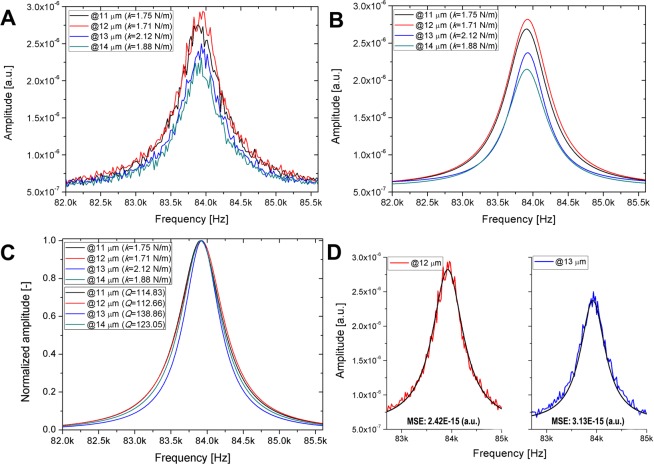
(**A**) Raw thermal noise spectra of the cantilever with 4 µm aperture size, directly exported from ARYA, obtained at four different laser spot positions (see Fig. 5B). (**B**) The fitted Lorentzian peak functions, also exported from ARYA. (**C**) The normalized peak functions at the same positions. (**D**) The raw thermal noise spectra and the fitted Lorentzian peak functions plotted together at two consecutive positions (MSE: mean squared error).

The spring constant is a fundamental attribute of the cantilever defined by material and structural properties, which theoretically should not be a function of laser beam spot position. Thus, the observed fluctuation in Fig. 5B must only originate from measurement errors. As discussed previously (Eq. 3), the Sader method requires the position and quality factor (resonance frequency/bandwidth) of the first fundamental resonance peak as input parameters, obtained from the thermal noise spectrum of the cantilever. It can be assumed that the variation of the calculated spring constant as functions of the laser position can primarily be attributed to the uncertainty of quality factor determination due to varying noise levels along the length of the cantilever. The presented thermal noise spectra of Fig. 7 prove this assumption.

Figure 7A presents four thermal noise spectra (directly exported from ARYA), measured in consecutive positions, corresponding to the data presented in Fig. 5B (cantilever aperture size: 4 µm). Figure 7B shows the fitted Lorentzian peak functions, also exported from ARYA. The spring constants calculated by the software are also given in the figures. Their variation is high, e.g. the min-max difference between the two consecutive positions of @12 and @13 µm is 0.41 N/m, which is a 20% relative difference. It can be seen in Fig. 7A that the obtained spectra are quite noisy. The peak positions do not vary much and thus the resonance frequencies are identified correctly (variation below 7 Hz), but the noise clearly affects the determination of bandwidth, defined as the full width at half maximum (FWHM). In Fig. 7C the manually normalized amplitude spectra from Fig. 7B are given, along with the quality factors (*Q*), calculated by ARYA. The data confirm that the variation of the spring constants originate from the determination of FWHM, as illustrated in Fig. 7D in the two consecutive positions. Both spectra are noisy, but while in the case of @12 µm the Lorentzian peak function is fitted on the outer boundary of the noisy edges, at @13 µm the fitted curve follows the inner boundary. This results in a smaller FWHM in the second case and leads to higher spring constant in this position. The error of fit (calculated as mean squared error (MSE) between the raw data and the Lorentzian fit, also given in Fig. 7D) is smaller for @12 µm, thus the obtained spring constant can be considered more reliable in this position. For a detailed analysis on the dependence of the calculated spring constant on the measured *ω*_R_ and FWHM please see the Supplementary Information (S1). The error of the built-in peak evaluation function is also compared with a manual peak evaluation in S2.

**Figure 8:**
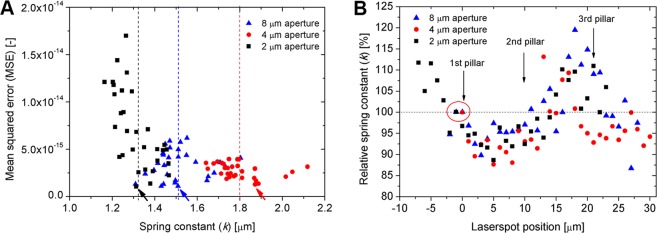
(**A**) Mean squared error (MSE) calculated between the raw thermal spectrum and its Lorentzian fit, as functions of the calculated spring constant (data corresponds to Fig. 5B). The arrows indicate the selected reference values used for rescaling the datasets, as presented in Fig. 8B. The dashed lines represent the mean of each distribution. (**B**) Relative spring constant values as functions of the laser position. The following values were used as a reference (100%) for calculations: 1.51 N/m for 8 µm, 1.87 N/m for 4 µm and 1.31 N/m for 2 µm aperture, respectively.

It was shown in Fig. 5B that the determined spring constants have a high variation, depending on the position of the laser spot along the cantilever and to identify which values can be considered acceptable we have plotted the mean squared error (MSE) of the Lorentzian peak function fits (performed by ARYA) in Fig. 8A as functions of the calculated spring constant values. This MSE can be considered to characterize the level of noise, which is superposed on the thermal spectra along the cantilever.

It is important to note, that the spring constant values calculated at the positions of smallest error coincide well with the mean of their distributions for the 2 and 8 µm apertures (Fig. 8A). For the cantilever with 4 µm aperture, the location of smallest error as functions of the spring constant is also well defined. In Fig. 8B these three values marked by arrows were used as a reference (100%) to calculate the relative spring constant values as functions of the laser spot position.

It can be seen that the separate curves in Fig. 5B now fit onto each other in a definite trend. The first intersection of this common trend with the 100% line (dashed in Fig. 8B) falls firmly to the first pillar (0 µm, marked with a red circle) and although the subsequent intersections also roughly coincide with the second (10 µm) and third (20 µm) pillars, these intersections are blurred and here the introduced error can be much higher. (Please note, that the diameter of a pillar is 3 µm and the precision of the given positions are around 1 µm).

### Calibration strategy

The presented data in the previous section can be used to devise a calibration strategy for the hollow FluidFM cantilevers. Although it is clear that the measured fluctuations of both InvOLS and spring constant are quite high (around ±30% for InvOLS (Fig. 5A) and −13/+20% for spring constant (Fig. 8B), their meaning and significance are quite different, and thus they should be treated differently.

*InvOLS* characterizes the local deflection of the cantilever. A lower InvOLS means higher force sensitivity, but the user does not necessarily have to minimize InvOLS to have reliable data. If determined by proper calibration at any position (e.g. the deflection-curve in the measurement spot is noise-free and the linear fit is correct) it can be used to scale the subsequently measured deflection-curves (Eq. 1) regardless of its absolute value, without introducing error to the gathered data. The only common mistake a user can do is to use a “general” InvOLS for a cantilever instead of measuring the local InvOLS at the exact position of the laser spot. As an example: the average value of InvOLS, considering the whole length of the cantilever is around 2.3 µm/V (Fig. 4). If one uses this as a general value for their cantilevers then places the laser on top of the first pair of pillars on a cantilever with 2 µm aperture, an error of −30% will directly be introduced for all the subsequently measured force data. A general rule of InvOLS calibration is that one must always use the local InvOLS of the hollow cantilever and must take care not to modify the position of the laser spot between calibration and measurements.

The *spring constant* is a completely different matter. As discussed previously this is a fundamental parameter of the cantilever which should not vary as functions of the position of the laser. The determination of the real spring constant for a cantilever is necessary: even for much simpler traditional AFM cantilevers the real spring constant can differ significantly from the nominal value given by the manufacturer, due to technological deviations. Our presented data in Fig. 8A also confirmed that the real spring constant of the three used cantilevers (considering the values obtained with the smallest error of Lorentzian fitting on the thermal spectra as ‘real’ spring constant values) were significantly different from their nominal values of 2 N/m (1.51 N/m for 8 µm, 1.87 N/m for 4 µm and 1.31 N/m for 2 µm aperture sizes, respectively). (Note: these obtained differences in the measured spring constants originate from technological variations and not characteristic for the aperture sizes.) The real problem is that as we demonstrated in Fig. 8B, calibrating the spring constant in the wrong position can result in a −13/+20% added error, and at this point the reliability of calibration (or the error of Lorentzian fit) cannot be directly assessed in the ARYA software.

Based on the presented data and previous discussions we advise the following *calibration strategy*:

1. *A good spot for reliable spring constant calibration is on the first pillars*.
2. *It is advised to move to the third pillar after spring constant calibration*, *calibrate the InvOLS here and perform the measurements*. The general noise originating from the laser interference is smaller here.
3. *For the most reliable spring constant calibration we advise to position the laser on the first pair of pillars*, *then move the laser around in a ± 2–3 µm range and repeat the calibration in 1 µm steps*. Although the goodness of Lorentzian fit on the thermal spectra is not displayed in the software, the raw and fitted curves can be exported and the mean squared error can be calculated manually to find the position where the noise is the smallest and thus the displayed spring constant is the most reliable.
4. *The InvOLS can be measured locally*, *but make sure not move the laser afterwards* (between calibration and subsequent measurements).

## Conclusions

It was demonstrated that due to the special structure of hollow FluidFM micropipette cantilevers the obtainable inverse optical lever sensitivity (InvOLS) and spring constant values depend significantly on the position of the laser spot along the length of the cantilever during calibration. It was shown that these fluctuations can be directly connected to the position of the pillars inside the hollow structure. The amount of these variations were around ±30% for InvOLS and −13/+20% for the spring constant measured the first 40 µm of the cantilever. Since these values directly scale and influence both the measured force and elastic modulus during single-cell force spectroscopy (SCFS) measurements a strategy for their precise calibration is advised. An optimal position for spring constant calibration was found to be on the first set of pillars, but considering the general noise superposed on the force-curves it is recommended to move to the third pillars afterwards to perform the InvOLS calibration and subsequent measurements. Checking the goodness of the Lorentzian peak function’s fit is also advised as an indicator for judging the reliability of the obtained spring constant values. It was also confirmed that using the polystyrene plate for InvOLS calibration does not introduce any significant error compared to using a silicon wafer for this purpose.

## Supplementary information

Supplementary information

## Data Availability

The datasets generated during and/or analyzed during the current study are available in the Mendeley repository, https://data.mendeley.com/datasets/zryrk4hy3j/1.
