# Publisher Correction: Spring constant and sensitivity calibration of FluidFM micropipette cantilevers for force spectroscopy measurements

**DOI:** 10.1038/s41598-019-54634-9

**Published:** 2019-11-26

**Authors:** Ágoston G. Nagy, Judit Kámán, Róbert Horváth, Attila Bonyár

**Affiliations:** 1 Nanobiosensorics Laboratory, Institute of Technical Physics and Materials Science, Centre for Energy Research, Hungarian Academy of Sciences, Budapest, Hungary; 2 Department of Electronics Technology, Budapest University of Technology and Economics, Budapest, Hungary

Correction to: *Scientific Reports* 10.1038/s41598-019-46691-x, published online 16 July 2019

This Article contains an error in the order of the Figures. Figures 5 and 6 were published as Figures 6 and 5 respectively. The correct Figures 5 and 6 appear below as Figures 1 and 2. The Figure legends are correct.

**Figure 1:**
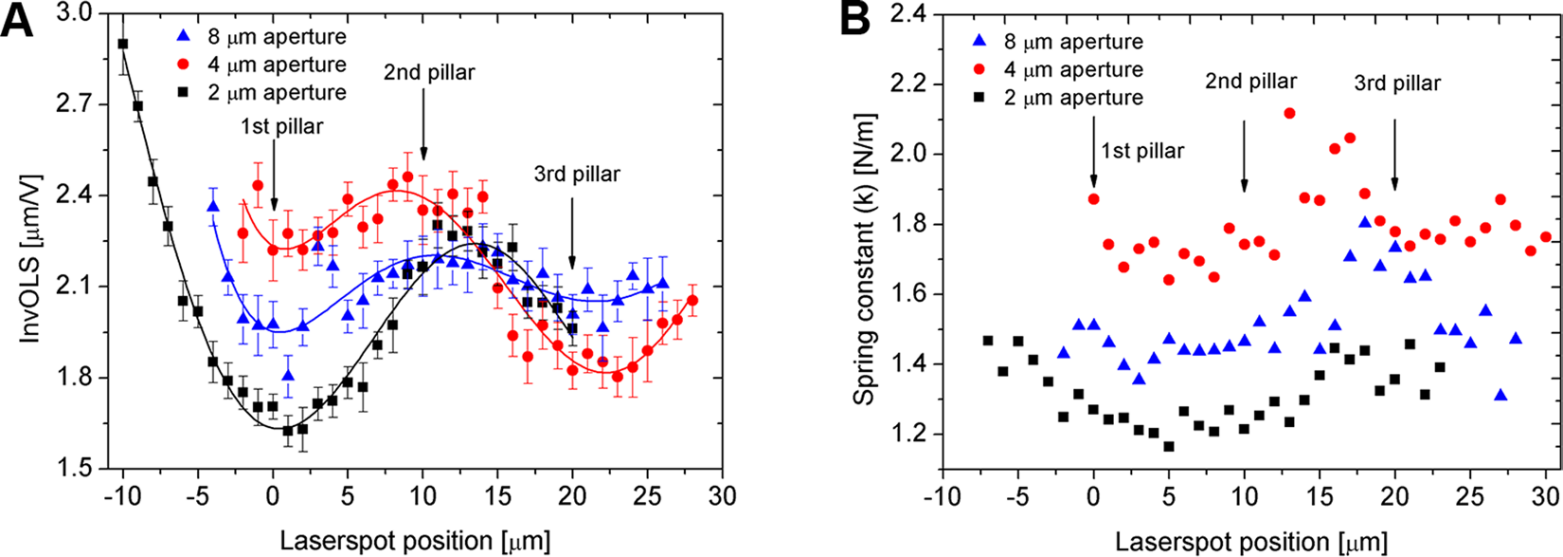
(**A**) The obtained InvOLS values measured in 1 μm steps on the polystyrene substrate with the three FluidFM cantilevers. The curves represent a 5th grade polynomial fit on the datasets. (**B**) The spring constant measured in 1 μm steps in air with the three FluidFM cantilevers, as functions of the laser spot position.

**Figure 2:**
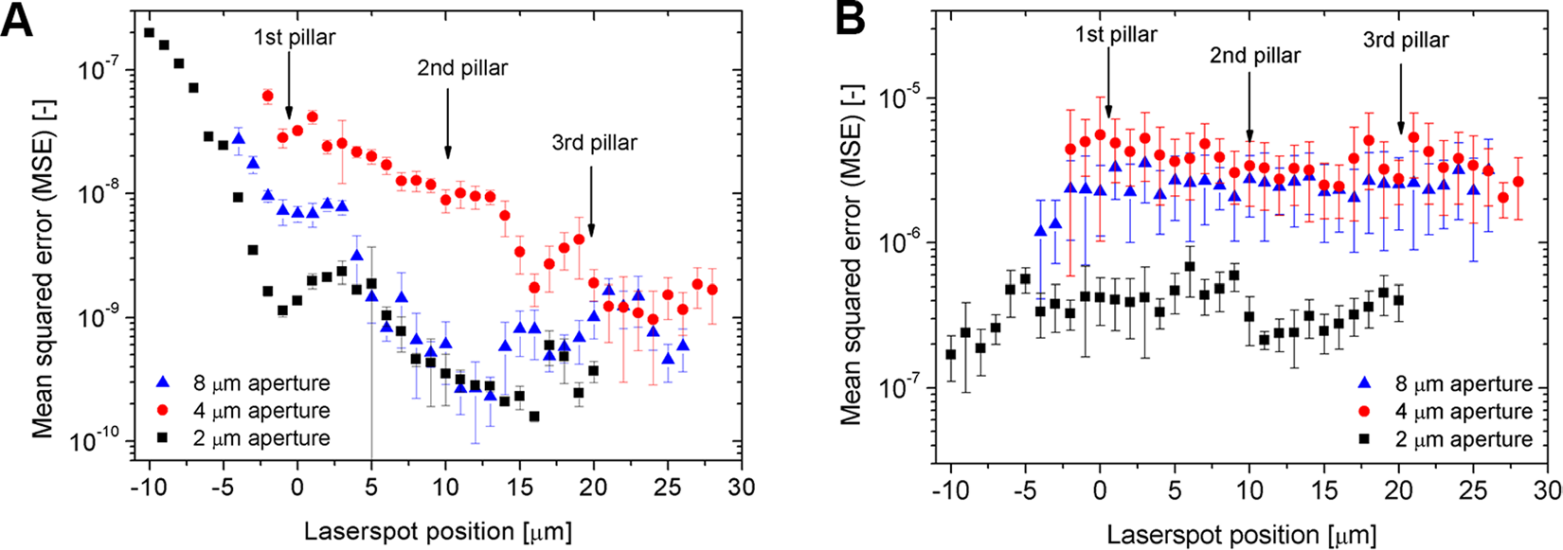
(**A**) The noise levels calculated as mean squared error (MSE) of linear fit on the baseline of a forcecurve (see Fig. 1B). (**B**) The same MSEs, measured on the linear indentation (approach) section of the forcecurve, which was used for InvOLS calculations.

