# Supplementary material for: Spring constant and sensitivity calibration of FluidFM micropipette cantilevers for force spectroscopy measurements
Source: Sci Rep. 2019 Jul 16;9:10287. doi: 10.1038/s41598-019-46691-x (PMC6635487; doi:10.1038/s41598-019-46691-x)
Supplement: Supplementary file 1 — Supplementary information [file 41598_2019_46691_MOESM1_ESM.pdf]

Supplementary information

Spring constant and sensitivity calibration of FluidFM micropipette cantilevers for force spectroscopy measurements

Ágoston G. Nagy[1,2], Judit Kámán[2], Róbert Horváth[1], Attila Bonyár[2*]

[1] Nanobiosensorics Laboratory, Institute of Technical Physics and Materials Science, Centre for Energy Research, Hungarian Academy of Sciences, Budapest, Hungary

[2] Department of Electronics Technology, Budapest University of Technology and Economics, Budapest, Hungary

Corresponding author:

Attila Bonyár, bonyar@ett.bme.hu, Tel.: 003614632758

S.1. The influence of FWHM and ω_R on the obtained spring constants

For the determination of the spring constant (*k*) the Sader method (Eq. S1) uses the resonance frequency (ω_R) and FWHM, obtained from the thermal noise spectrum of a cantilever. As we have demonstrated in Fig. 7 the resonance peak of this spectrum is quite noisy and both the determination of ω_R and FWHM has some variation. We stated that this variation is much smaller (in relative terms) for ω_R compared to FWHM, but since *k* depends on the third power of ω_R, we have plotted these dependencies in Fig. S1. The data proves that the obtained value of *k* is insensitive to ω_R, while it shows a clear negative correlation with FWHM. These values were gained with the built-in peak evaluation function of the FluidFM.

$$k = 0.1906\rho w^2 LQ\Gamma_i(\omega_R)\omega_R^2 = 0.1906\rho w^2 L\Gamma_i(\omega_R)\omega_R^3 FWHM^{-1} \quad \text{(S1)}$$

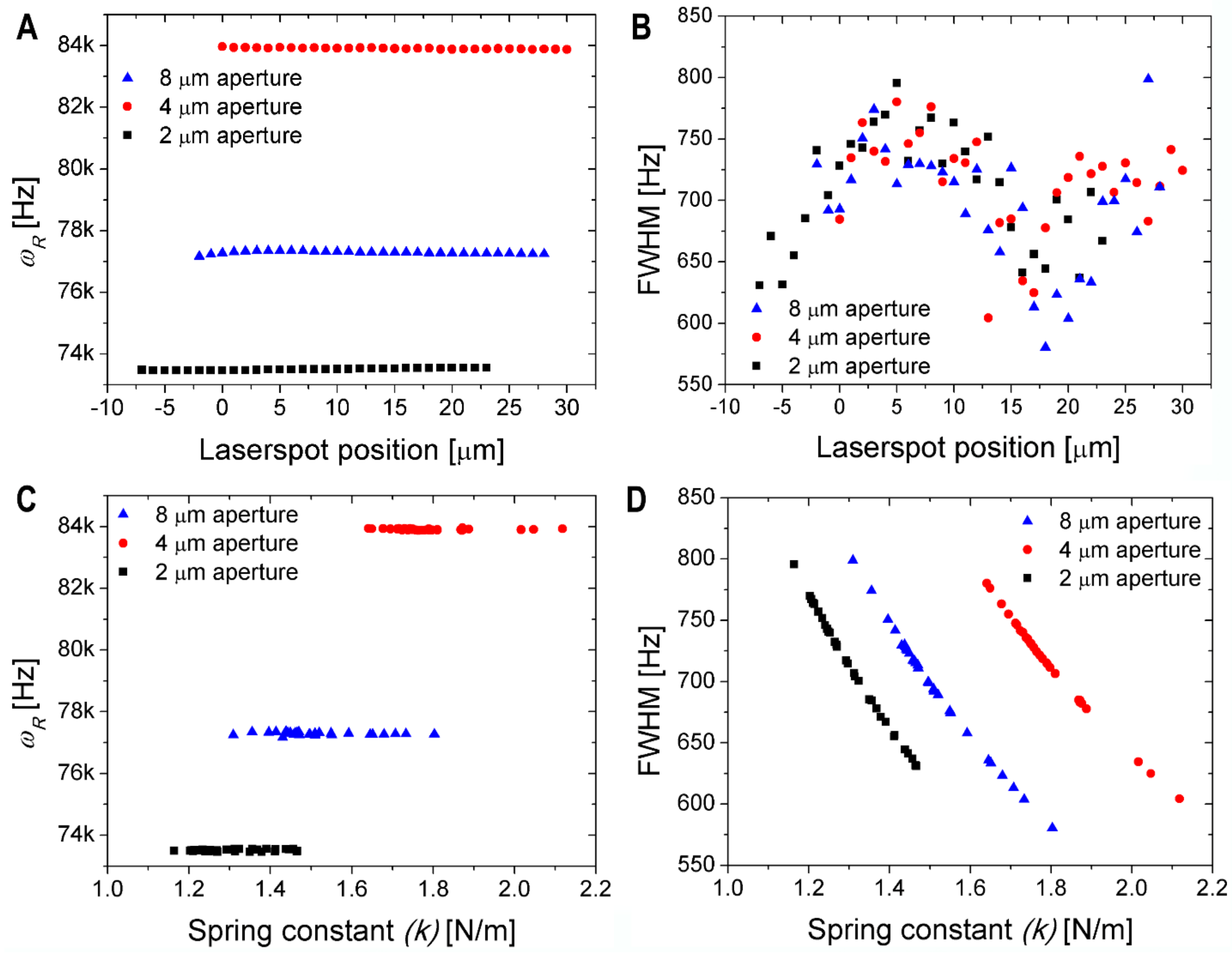

Figure S1. A) The obtained resonance frequencies in function of the position of the laser spot. **B)** The obtained FWHM-s in function of the position of the laser spot. **C)** Correlation between the measured resonance frequencies and the calculated spring constant. **D)** Correlation between the measured FWHM and the calculated spring constant.

S.2. Comparison of built-in and manual resonance peak evaluation

To characterize the error of the built-in peak evaluation software of FluidFM we have manually fitted the exported raw thermal noise spectra and compared our results with the results of ARYA. A Lorentzian peak function was fitted on the resonance peak with Origin software, using the “Fit till converged” option, which results in the smallest possible error of fit. The results are compared in Fig. S2 for the first 15 points of the cantilever with 4 μm aperture.

It can be seen, that even the ‘best possible’ manual fit with Origin resulted in the same characteristics, in function of laser spot’s position. Although the deviation of the manual fit is somewhat smaller, it was not possible to filter out the effect of the noise completely. More importantly, the mean squared errors of both fits resulted in the same characteristics, which are also in good agreement with Fig. 8B and also with Fig. S1.

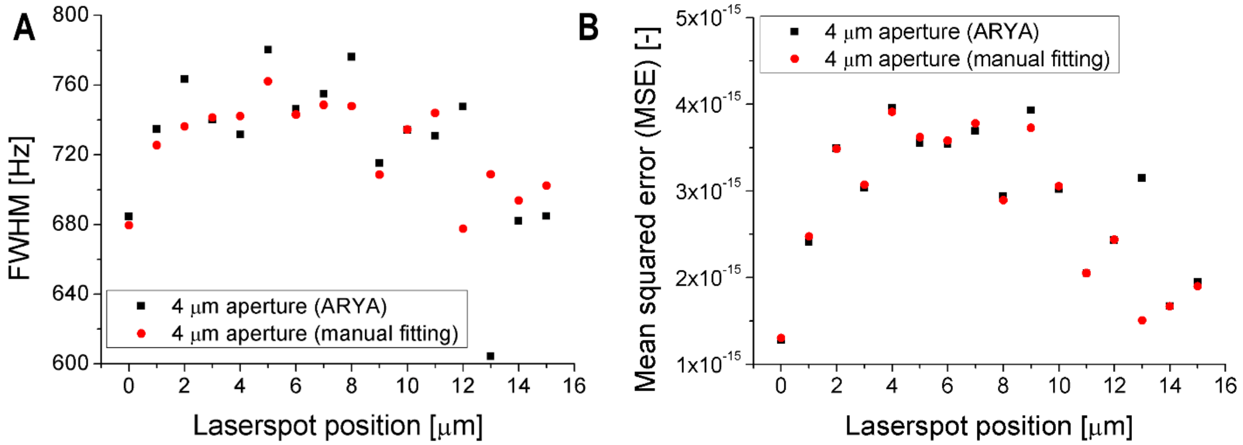

Figure S2. A) The FWHM values obtained with the built-in Lorentzian peak witting (within the ARYA software of the FluidFM) compared to manual fitting on the raw thermal spectra. **B)** The mean squared errors of both methods in function of the laser spot’s position.
